# Non-coding RNAs are involved in tumor cell death and affect tumorigenesis, progression, and treatment: a systematic review

**DOI:** 10.3389/fcell.2024.1284934

**Published:** 2024-02-28

**Authors:** Zeping Han, Wenfeng Luo, Jian Shen, Fangmei Xie, Jinggen Luo, Xiang Yang, Ting Pang, Yubing Lv, Yuguang Li, Xingkui Tang, Jinhua He

**Affiliations:** ^1^ Central Laboratory, Guangzhou Panyu Central Hospital, Guangzhou, China; ^2^ Rehabilitation Medicine Institute of Panyu District, Guangzhou, China; ^3^ Department of General Surgery, Guangzhou Panyu Central Hospital, Guangzhou, China; ^4^ Department of Gynaecology and Obstetrics, Guangzhou Panyu Central Hospital, Guangzhou, China; ^5^ Clinical Laboratory, Guangzhou Panyu Central Hospital, Guangzhou, China; ^6^ He Xian Memorial Hospital, Southern Medical University, Guangzhou, China

**Keywords:** non-coding RNAs, autophagy, ferroptosis, pyroptosis, tumorigenesis, tumor progression, treatment

## Abstract

Cell death is ubiquitous during development and throughout life and is a genetically determined active and ordered process that plays a crucial role in regulating homeostasis. Cell death includes regulated cell death and non-programmed cell death, and the common types of regulatory cell death are necrosis, apoptosis, necroptosis, autophagy, ferroptosis, and pyroptosis. Apoptosis, Necrosis and necroptosis are more common than autophagy, ferroptosis and pyroptosis among cell death. Non-coding RNAs are regulatory RNA molecules that do not encode proteins and include mainly microRNAs, long non-coding RNAs, and circular RNAs. Non-coding RNAs can act as oncogenes and tumor suppressor genes, with significant effects on tumor occurrence and development, and they can also regulate tumor cell autophagy, ferroptosis, and pyroptosis at the transcriptional or post-transcriptional level. This paper reviews the recent research progress on the effects of the non-coding RNAs involved in autophagy, ferroptosis, and pyroptosis on tumorigenesis, tumor development, and treatment, and looks forward to the future direction of this field, which will help to elucidate the molecular mechanisms of tumorigenesis and tumor development, as well as provide a new vision for the treatment of tumors.

## Background

Cell death is an irreversible process that plays an important role in normal development and inhibits the rapid growth of tumor cells (Mahapatra et al., 2021; Patra et al., 2022). Cell death includes regulatory cell death and non-programmed cell death. Regulatory cell death is ubiquitous during development and is an active and orderly process determined by genes that plays an important role in maintaining homeostasis (Chen L. et al., 2021). Non-coding RNAs are RNA molecules that do not encode proteins, including mainly microRNAs (miRNAs), long non-coding RNAs (lncRNAs), and circular RNAs (circRNAs). Non-coding RNAs participate in protein, DNA, and RNA interactions and have roles in a variety of cellular activities, including gene activation and silencing, RNA splicing, modification, and editing, and protein translation (Bahreini et al., 2021). An increasing number of studies have demonstrated that non-coding RNAs regulate tumor cell death by acting directly on cell death-related proteins or acting indirectly on downstream targets or pathways. In recent years, tremendous progress has been made in the study of the molecular mechanisms underlying the regulation of tumor cell death by non-coding RNAs, which is of great significance for elucidating the mechanisms of tumorigenesis and tumor development and guiding the treatment and intervention of tumors based on cell death. This article reviews the progress of research from this aspect.

## Classification and regulatory mechanisms of cell death

At present, the common types of regulatory cell death are autophagy, ferroptosis, and pyroptosis (Buchser et al., 2012; Green, 2019). The differences in these processes are shown in Supplementary Table S1 (Additional File S1). Autophagy is a highly conserved self-digestion process in eukaryotic cells that extensively regulates cell growth, development, senescence, and death. In some cases, autophagy acts on cell death as autophagic cell death. Autophagy is the process by which cells use lysosomes to degrade damaged organelles and macromolecular substances to maintain homeostasis. The cells first form a monolayer or bilayer membrane that develops into a vesicle-like autophagosome, which then fuses with a lysosome to form an autolysosome, leading to the lysosomal hydrolase degradation of the contents and the recycling of the products (New and Tooze, 2019; Chen et al., 2022), as shown in Supplementary Figure S1 (Additional File S2).

Ferroptosis is a newly identified form of regulatory cell death caused by the abnormal accumulation of lipid reactive oxygen species (ROS). Ferroptosis and iron metabolism, glutathione metabolism, and lipid peroxidation are closely related, Thus, a large number of molecules have roles in ferroptosis, including transferrin receptor 1, ferritin, cystine/glutamate reverse transporter, glutathione peroxidase 4 (GPX4), and lipoxygenase (Rochette et al., 2022). The regulatory mechanisms of ferroptosis are described in Supplementary Figure S2 (Additional File S3).

Pyroptosis is a novel form of programmed cell death that depends mainly on the cleavage and activation of gasdermin protein by the inflammasome-activating caspase family of proteins, which then translocates to the membrane to form holes, leading to cell swelling, cytosolic outflow, and cell membrane rupture (Zhang et al. 2021c). The regulatory mechanism of pyroptosis is shown in Supplementary Figure S3 (Additional File S4).

## Features and functions of non-coding RNAs

Non-coding RNAs are transcribed, but are not translated into a protein, and include mainly miRNAs, lncRNAs, and circRNAs (Bahreini et al., 2021). The features and functions of miRNAs, lncRNAs, and circRNAs are summarized in Supplementary Table S2 (Additional File S1). miRNAs are an endogenous class of RNA molecules that are 21–25 nucleotides in length. miRNAs regulate various metabolic pathways at the transcription and translation levels and have key roles in regulating tumor cell growth, migration, invasion, and chemoresistance (Ambros, 2001). lncRNAs are RNA molecules greater than 200 nucleotides in length, with or without an open reading frame, that are involved in transcriptional silencing and activation, chromosome modification, and nuclear transport (Anastasiadou et al., 2018). circRNAs are non-coding RNA molecules formed by reverse splicing that are stable and not easy to degrade. circRNAs are derived from introns or exons, can act as RNA sponges, and can also be combined with proteins, so as to participate in the regulation of gene expression and affect protein function (Zheng et al., 2017).

## Role of miRNAs in tumor cell death for tumorigenesis, development, and treatment

### miRNAs and autophagy

In recent years, miRNAs have been shown to be involved mainly in influencing the role of autophagy in lung cancer, multiple myeloma, kidney cancer, oral cancer, liver cancer, nasopharyngeal carcinoma (NPC), melanoma, thyroid cancer, and prostate cancer (PCa), as summarized in Table 1. For example, miRNA-153-3p expression is low in gefitinib-resistant non-small cell lung cancer (NSCLC) cell lines, and its expression level is negatively correlated with that of the autophagic activity marker tubulin 1 light chain 3. miRNA-153-3p overexpression inhibits the occurrence of autophagy and enhances sensitivity to gefitinib by downregulating the expression of autophagy-related 5 homolog (ATG5) (Zhang et al., 2019a). The expression levels of miRNA-125b and miRNA-143 are significantly correlated with the concentrations of beta-2-microglobulin, albumin, and hemoglobin. The upregulation of miRNA-125b and downregulation of miRNA-143 both promote autophagy, thus inhibiting apoptosis in myeloma cells (Shang et al., 2018). miR-30a inhibits autophagy in renal cancer cells by downregulating the expression of Beclin-1 and interferes with sorafenib-mediated cell apoptosis through an autophagy-dependent pathway (Zheng et al., 2015). Isoliquiritigenin promotes apoptosis and autophagy in oral squamous cell carcinoma (OSCC) cells by downregulating the expression of miRNA-21 and miRNA-155, which provides new targets for the treatment of OSCC (Mohamed et al., 2022). Mitogen-inducible gene 6 (Mig-6) promotes apoptosis and autophagy in hepatocellular carcinoma (HCC) cells by regulating the expression of miR-193a-3p (Qu et al., 2022). miR-1278 specifically regulates the expression of ATG2B to promote autophagy in NPC cells, thereby increasing the chemical resistance of NPC cells to cisplatin; miR-1278 may become a novel therapeutic target for the treatment of NPC (Zhao et al., 2020). miR-30a-5p inhibits ATG5-mediated autophagy in lung SCC cells and slows the progression of lung SCC through the autophagy pathway (Yang et al., 2021b). miR-18a-5p expression is significantly increased in melanoma tissues and cell lines, and the expression of ephrin type-A receptor 7 (EPHA7) is negatively regulated to promote the proliferation of melanoma cells and inhibits apoptosis and autophagy, which provide clues to reveal the pathogenesis of miRNA-mediated melanoma (Guo Y. et al., 2021). miR-216a expression is downregulated in colorectal cancer (CRC) tissues, and autophagy is inhibited by the transforming growth factor beta 1 (TGF-β)/microtubule-associated protein 1S (MAP1S) pathway (Wang Y. et al., 2019). The high expression of miR-221/222 is associated with the regional lymph node and distant metastasis stage of papillary thyroid carcinoma, and miR-221/222 target ATG10 expression, thus inhibiting autophagy and apoptosis in papillary thyroid carcinoma cells (Shen et al., 2020). In PCa cells, the upregulation of miR-381 expression suppresses the expression of recombinant reelin and then suppresses the activation of the PI3K/AKT/mammalian target of rapamycin (mTOR) signaling pathway to promote apoptosis and autophagy (Liao and Zhang, 2020).

**TABLE 1:** miRNAs and autophagy.

Disease	miRNA	Mechanism	Ref.
NSCLC	miRNA-153-3p	miRNA-153-3p downregulates ATG5 and inhibits autophagy to enhance the sensitivity of gefitinib in NSCLC	Zhang et al. (2019a)
MM	miRNA-125b/143	miRNA-125b and miRNA-143 upregulate autophagy to inhibit the apoptosis of myeloma cells	Shang et al. (2018)
RCC	miR-30a	miR-30a dysregulation interferes with sorafenib-mediated apoptosis through autophagy-dependent pathways	Zheng et al. (2015)
OSCC	miRNA-21/155	Downregulation of miRNA-21 and miRNA-155 expression promotes autophagy in OSCC cells	Mohamed et al. (2022)
HCC	miR-193a-3p	Mig-6 affects autophagy by modulating miR-193a-3p expression	Qu et al. (2022)
NPC	miR-1278	miR-1278 promotes autophagy by regulating ATG2B expression	Zhao et al. (2020)
SCC of the lung	miR-30a-5p	miR-30a-5p slows the progression of SCC of the lung through autophagy pathways	Yang et al. (2021b)
Melanoma	miR-18a-5p	miR-18a-5p regulates EPHA7 expression to trigger autophagy	Guo Y. et al. (2021)
CRC	miR-216a	miR-216a regulates the TGF-β/MAP1S pathway to inhibit autophagy	Wang Y. et al. (2019)
PTC	miR-221/222	miR-221/222 regulate ATG10 expression to inhibit autophagy in papillary thyroid carcinoma cells	Shen et al. (2020)
PCa	miR-381	The miR-381/PI3K/AKT/mTOR pathway promotes autophagy	Liao and Zhang (2020)

### miRNAs and ferroptosis

Studies of miRNAs involved in the effects of ferroptosis on the occurrence, development, and treatment of lung cancer, melanoma, CRC, ovarian cancer, HCC, OSCC, PCa, osteosarcoma, and breast cancer are summarized in Table 2. For example, miRNA-101-3p is downregulated in lung adenocarcinoma, and miR-101-3p and the lncRNA G-tetra-chain body formation sequence (GSEC) regulate each other to promote ferroptosis in lung adenocarcinoma cells (Jiang et al., 2021). miR-27a-3p directly targets the expression of solute carrier family 7 member 11 (SLC7A11) in NSCLC cells to regulate ferroptosis (Lu et al., 2021a). miR-6077 regulates KEAP1-NRF2(nuclear factor erythroid 2-related factor)-SLC7A11-NQO1 (NAD (P)H quinone dehydrogenase 1)-mediated ferroptosis to protect lung adenocarcinoma cells from cisplatin/pemetrexed-induced cell death, thereby increasing their resistance to chemotherapeutic drugs (Bi et al., 2022). In melanoma cells, miR-9 acts as an important regulator of ferroptosis, and its overexpression inhibits glutamic-oxaloacetic transaminase 1 (GOT1) expression and reduces ferroptosis induced by erastin and RAS-selective lethal 3 (RSL3), an inhibitor of GPX4 (Zhang et al., 2018). miR-137 negatively regulates ferroptosis by directly targeting the binding glutamine transporter SLC1A5 in melanoma cells, pointing the way for potential therapies for melanoma (Luo et al., 2018). miR-19a promotes the proliferation and invasion of CRC cells and suppresses ferroptosis by inhibiting the expression of iron-response element-binding protein 2 (IREB2) (Fan et al., 2022). Upregulation of miR-424-5p suppresses acyl-CoA synthetase long chain family member 4 (ACSL4) expression, thereby reducing ferroptosis induced by erastin and RSL3, while downregulation of miR-424-5p increases the sensitivity of ovarian cancer cells to erastin and RSL3 (Ma et al., 2021). The expression of miR-3200-5p, which binds to activated transcription factor 4 (ATF4), regulates ferroptosis and inhibits the proliferation and metastasis of HCC cells (Guan et al., 2022). The ETS proto-oncogene 1, transcription factor (ETS1)/miR-23a-3p/ACSL4 axis increases the resistance of liver cancer cells to sorafenib by regulating ferroptosis, and miR-23a-3p may be a potential target for improving the sensitivity of HCC to sorafenib (Lu et al., 2022). miR-142-3p binds to SLC7A5 and promotes ferroptosis in hepatitis B virus-infected M1 macrophages, thereby accelerating the progression of HCC (Hu et al., 2022). In the OSCC SCC-25 cell line, miR-34c-3p overexpression suppresses cell proliferation and increases ROS, malondialdehyde (MDA), and iron concentrations, and decreases glutathione and GPX4 expression levels to promote ferroptosis (Sun et al., 2022). miR-15a overexpression and decreased GPX4 expression inhibit the proliferation of PCa cells and increase intracellular iron and ROS accumulation, and miR-15a induces ferroptosis by regulating GPX4 expression (Xu et al., 2022). The upregulation of miR-1287-5p expression increases the sensitivity of osteosarcoma cells to cisplatin chemotherapy by inhibiting GPX4 expression (Xu Z. et al., 2021). The ectopic expression of SLC7A11 partially reverses miR-5096-mediated ROS, lipid peroxidation, iron accumulation, GSH, hydroxyl radicals, mitochondrial membrane potential, and colony formation in breast cancer cells (Yadav et al., 2021).

**TABLE 2:** miRNAs and ferroptosis.

Disease	miRNA	Mechanism	Ref.
Lung cancer	miRNA-101-3p	Mutual regulation of miR-101-3p and lncRNA GSEC	Jiang et al. (2021), Lu et al. (2021a), Bi et al. (2022)
	miR-27a-3p	miR-27a-3 regulates SLC7A11 expression	
	miR-6077	miR-6077 targets CDKN1A-CDK1 expression	
Melanoma	miR-9	miR-9 targets the expression of GOT1	Zhang et al. (2018), Luo et al. (2018)
	miR-137	miR-137 inhibits glutamate breakdown	
CRC	miR-19a	miR-19a inhibits the expression of IREB2	Fan et al. (2022)
OC	miR-424-5p	miR-424-5p suppresses ACSL4 to regulate ferroptosis	Ma et al. (2021)
HCC	miR-3200-5p	miR-3200-5p binds to ATF4 to regulate ferroptosis	Guan et al. (2022), Lu et al. (2022), Hu et al. (2022)
	miR-23a-3p	ETS1/miR-23a-3p/ACSL4 axis regulates ferroptosis	
	miR-142-3p	miR-142-3p in combination with SLC3A2 promotes ferroptosis in hepatitis B virus-infected M1 macrophages	
OSCC	miR-34c-3p	miR-34c-3p increases ROS, MDA, and iron deposition and decreases glutathione and GPX4 expression levels	Sun et al. (2022)
PCa	miR-15a	miR-15a regulates GPX4 expression	Xu et al. (2022)
Osteosarcoma	miR-1287-5p	miR-1287-5p inhibits GPX4 expression	Xu Z. et al. (2021)
Breast cancer	miR-5096		Yadav et al. (2021)
		Ectopic expression of SLC7A11 reverses miR-5096-mediated iron accumulation	

### miRNAs and pyroptosis

Studies of the roles of miRNAs in pyroptosis for the development of osteosarcoma, neurocytoma, glioma, cervical cancer, and breast cancer are summarized in Table 3. For example, the downregulation of miR-181a expression in osteosarcoma cells activates the expression of nucleotide-binding oligomerization domain-like receptor pyrin domain-containing 3 (NLRP3) to promote pyroptosis and inhibit tumor growth (Tian et al., 2020). miR-195 expression is significantly downregulated in neuroblastoma SH-SY5Y cells infected with enterovirus A71, and miR-195 binds to NLR family member X1 and NLRs to regulate the pyroptosis induced by enterovirus A71 infection (Zhu X. et al., 2021). In glioma tissues as well as the glioblastoma cell lines U87 and T98G, caspase-1 expression is increased while miR-214 expression is significantly downregulated, and miR-214 regulates caspase 1-mediated pyroptosis to inhibit cell proliferation and migration, providing a novel intervention for glioma (Jiang et al., 2017). miR-214 expression is significantly downregulated in cervical cancer cell lines and regulates NLRP3 expression, which induces pyroptosis (Yu et al., 2020). miRNA-155-5p expression is upregulated in triple-negative breast cancer cells, while downregulation of its expression induces pyroptosis and enhances the role of cetuximab in the MDA-MB-468 xenograft model (Xu W. et al., 2021).

**TABLE 3:** miRNAs and pyroptosis.

Disease	miRNA	Mechanism	Ref.
Osteosarcoma	miR-181a	miR-181a downregulates NLRP3 expression	Tian et al. (2020)
Neurocytoma	miR-195	miR-195 regulates NLRX1 expression	Zhu X. et al., 2021
Glioma	miR-214	miR-214 regulates caspase-1 expression	Jiang et al. (2017)
Cervical cancer	miR-214	miR-214 regulates NLRP3 expression	Yu et al. (2020)
Breast cancer	miR-155-5p	NA	Xu W. et al. (2021)

## Role of lncRNAs in tumor cell death for tumorigenesis, development, and therapy

### lncRNAs and autophagy

lncRNAs are involved mainly in autophagy affecting uveal melanoma, CRC, gastric cancer, glioma, breast cancer, glioblastoma, glioma, hepatoma, HCC, NSCLC, bladder cancer, acute myelogenous leukemia (AML), chronic myelogenous leukemia (CML), ovarian cancer, PCa, thyroid papillary carcinoma, NPC, OSCC, osteosarcoma, prolactinoma, and pancreatic cancer, as summarized in Table 4. For example, the lncRNA ZNF706 neighboring transcript 1 (ZNNT1) is expressed at a low level in uveal melanoma cells, and ZNNT1 promotes autophagy by upregulating ATG12 expression, and thus has a tumor suppressive effect (Li et al., 2020).

**TABLE 4:** lncRNAs and autophagy.

Disease	lncRNA	Mechanism	Ref.
Uveal melanoma	ZNNT1	ZNNT1 upregulates ATG12 expression	Li et al. (2020)
CRC	HAGLROS	HAGLROS regulates the miR-100/ATG5 axis and PI3K/AKT/mTOR signaling pathway	Zheng et al. (2019)
	UCA1	NA	Song et al. (2019)
Gastric cancer	EIF3J-DT	EIF3J-DT regulates ATG14 expression	Luo et al. (2021)
Glioma	GAS5	GAS5 activates the mTOR signaling pathway	Huo and Chen (2019)
	TPT1-AS1	The TPT1-AS1/miR-770-5p/STMN1 axis mediates autophagy	Jia et al. (2020)
Breast cancer	H19	H19 induces autophagy through the SAHH/DNMT3B axis	Wang J. et al. (2019)
Neuroglioma	DRAIC	DRAIC downregulates GLUT1 expression, the target gene of NF-κB, and activates AMPK, thereby inhibiting mTOR expression	Saha et al. (2021)
	H19	H19 regulates the mTOR/Unc-51 axis	Zhao et al. (2021)
HCC	HAGLROS	HAGLROS regulates the miC-5095/ATG12 axis and PI3K/AKT/mTOR signaling pathway	Wei et al. (2019)
	MALAT1	MALAT1 regulates miR-146a expression	Peng et al. (2020)
Cervical cancer	HOTAIR	HOTAIR regulates the Wnt signaling pathway	Guo et al. (2019)
NSCLC	NBAT1	The NBAT1-PSMD10-ATG7 axis inhibits autophagy	Zheng et al. (2018)
Bladder cancer	SNHG1	The SNHG1/miR-493-5p/ATG14/pathway promotes autophagy	Guo C. et al. (2021)
AML	HOTAIRM1	Nuclear HOTAIRM1 promotes the formation of the MDM2-EGR1 complex to degrade EGR1, and cytoplasmic HOTAIRM1 downregulates miR-152-3p expression, thereby increasing ULK3 expression	Jing et al. (2021)
CML	OIP5-AS1	OIP5-AS1 regulates the miR-30e-5p/ATG12 axis	Dai et al. (2021)
Ovarian cancer	SNHG7	Metformin mediates autophagy by regulating SNHG7/miR-3127-5p	Yu et al. (2022)
PCa	PCDRlnc1	PCDRlnc1 interacts with UHRF1 leading to the activation of Beclin-1 signaling	Xie et al. (2022)
PCT	RP11-476D10.1	RP11-476D10.1 binds to miR-138-5p and promotes LRRK2 expression	Zhao et al. (2019)
NPC	HAGLROS	HAGLROS adsorbs miR-100 and regulates ATG14 expression	Zhang et al. (2019b)
OSCC	LINC01207	LINC01207 regulates the autophagy of miR-1301-3p/LDHA axis	Lu et al. (2021b)
Osteosarcoma	SNHG15	SNHG15 enhances autophagy by targeting miR-381-3p/GFRA1	Zhang J. et al. (2020)
Prolactinoma	CLRN1-AS1	FOXP1 induces ClRN1-AS1 expression and regulates the miR-217/DKK1/Wnt/β-catenin signaling pathway	Wang C. et al. (2019)
Pancreatic carcinoma	PVT1	PVT1 upregulates Pygo2 and ATG14 expression, thereby promoting autophagy through the miR-619-5p/Wnt/β-catenin signaling pathway	Zhou et al. (2020)

The high expression of the lncRNA HAGLR complementary chain (HAGLROS) is associated with the shortened survival time of patients with CRC; HAGLROS induces CRC cell apoptosis and suppresses autophagy through the miR-100/ATG5 and PI3K/AKT/mTOR pathways (Zheng et al., 2019). Downregulation of lncRNA urothelial cancer-associated 1 (UCA1) expression suppresses CRC cell proliferation and autophagy (Song et al., 2019). EIF3J divergent transcript (EIF3J-DT) is highly expressed in gastric cancer cells with chemotherapeutic drugs, and EIF3J-DT specifically regulates ATG14 expression, activates autophagy, and induces drug resistance in gastric cancer cells (Luo et al., 2021). The lncRNA growth arrest-specific 5 (GAS5) is expressed in the U138 and LN18 cell lines, where it induces cisplatin sensitivity through the activation of mTOR signaling and thereby inhibits autophagy (Huo and Chen, 2019). The expression of tumor protein translation control 1 antisense RNA 1 (TPT1-AS1) is upregulated in glioma cells, and the TPT1-AS1/miR-770-5p/stathmin 1 (STMN1) axis mediates autophagy in glioma cells, which is important for the targeted treatment of glioma (Jia et al., 2020). Downregulation of H19 imprinted maternally expressed transcript (H19) expression inhibits autophagy in tamoxifen-resistant MCF7 cells; H19 induces autophagy through the S-adenosyl-L-homocysteine hydrolase (SAHH)/DNA methyltransferase 3 beta (DNMT3B) axis, which helps to elucidate the molecular mechanism of tamoxifen resistance in breast cancer (Wang J. et al., 2019). Downregulated RNA in cancer (DRAIC). Inhibits the invasion of glioblastoma-derived cell lines and activates adenosine monophosphate-activated protein kinase (AMPK) by downregulating the expression of glucose transporter 1 (GLUT1), thereby inhibiting the expression of mTOR, and eventually leading to increased autophagy (Saha et al., 2021). Overexpression of H19 promotes the proliferation and migration of glioma cells, and H19 promotes the proliferation and autophagy of glioma cells through the mTOR/Unc-51-like autophagy-activating kinase 1 (ULK1) pathway (Zhao et al., 2021). HAGLROS is highly expressed in HCC, and its high expression may be related to both the miC-5095/ATG12 axis and the PI3K/AKT/mTOR signaling pathway, which are involved in apoptosis and autophagy (Wei et al., 2019). Downregulation of the expression of metastasis-related lung adenocarcinoma transcript 1 (MALAT1) promotes apoptosis and autophagy in HCC cells and regulates miR-146a expression, affecting the progression of HCC (Peng et al., 2020). Upregulation of HOX transcript antisense RNA (HOTAIR) expression promotes the progression of cervical cancer, while its downregulation reduces autophagy and reverses epithelial stromal transformation by inhibiting the Wnt signaling pathway, thereby enhancing the sensitivity of cervical cancer to radiotherapy (Guo et al., 2019). Downregulation of neuroblastoma-associated transcript 1 (NBAT1) expression inhibits autophagy in NSCLC cells. Furthermore, the interaction of NBAT1 with proteasome 26S subunit, non-ATPase 10 (PSMD10) promotes autophagic degradation (Zheng et al., 2018). High small nucleolar RNA host gene 1 (SNHG1) expression is positively correlated with bladder cancer cell invasion, proliferation, and autophagy, and SNHG1 functions through the miR-493-5p/ATG14 pathway (Guo C. et al., 2021). HOXA transcript antisense RNA, myeloid-specific 1 (HOTAIRM1) promotes the proliferation and autophagy of AML cells; nuclear HOTAIRM1 promoted early growth response 1 (EGR1) degradation by serving as a scaffold to facilitate MDM2 (Murine double minute 2)-EGR1 complex formation, while cytoplasmic HOTAIRM1 acted as a sponge for miR-152-3p to increase ULK3 (Unc-51 like kinase 3) expression (Jing et al., 2021). In CML K562 cells, upregulation of opa interacting protein 5 antisense RNA 1 (OIP5-AS1) expression enhances autophagy, while its downregulation suppresses autophagy and enhances sensitivity to imatinib, and OIP5-AS1 promotes autophagy-related imatinib resistance in CML cells through the miR-30e-5p/ATG12 axis (Dai et al., 2021). Metformin inhibits the viability of paclitaxel ovarian cancer cells, increases the expression of SNHG7, and promotes autophagy in ovarian cancer cells; Metformin enhances the sensitivity of ovarian cancer cells to paclitaxel by regulating the SNHG7/miR-3127-5p axis to mediate autophagy (Yu et al., 2022). Decreasing PCa docetaxel resistance-associated lncRNA1 (PCDRlnc1) expression significantly inhibits autophagy in PCa cells; PCDRlnc1 interacts with ubiquitin-like with plant homeodomain and ring finger domain 1 (UHRF1) and promotes its transcription in PCa cells, leading to the activation of autophagy-related Beclin-1 protein (Xie et al., 2022). Silencing of lncRNA RP11-476D10.1 expression enhances apoptosis and autophagy in papillary thyroid carcinoma cells; lncRNA RP11-476D10.1 binds to miR-138-5p and promotes leucine-rich repeat kinase 2 (LRRK2) expression (Zhao et al., 2019). Silencing of HAGLROS expression promotes NPC cell apoptosis and inhibits autophagy in NPC cells; HAGLROS affects NPC progression by regulating ATG14 expression by adsorbing miR-100 (Zhang et al., 2019b). LINC01207 expression is upregulated in OSCC cells to promote apoptosis and autophagy; the LINC01207/miR-1301-3p/lactate dehydrogenase A (LDHA) regulatory axis promotes the proliferation of OSCC cells (Lu et al., 2021b). SNHG15 is upregulated in doxorubicin-resistant cell lines, and knockdown of SNHG15 expression inhibits the proliferation and autophagy of osteosarcoma cells; SNHG15 targets miR-381-3p/GDNF family receptor alpha 1 (GFRA1) expression to promote autophagy and enhances the resistance of osteosarcoma cells to doxorubicin (Zhang J. et al., 2020). CLRN1 antisense RNA 1 (CLRN1-AS1) inhibits the proliferation and autophagy of prolactinoma cells; forkhead box protein P1 (FOXP1) induces the expression of CLRN1-AS1, which adsorbs miR-217 and affects the biological function of pituitary prolactinoma cells through the Dickkopf WNT signaling pathway inhibitor 1 (DKK1)/Wnt/β-catenin signaling pathway (Wang C. et al., 2019). Pvt1 oncogene (PVT1) is upregulated in gemcitabine-resistant pancreatic cancer cell lines, and PVT1 promotes Pygopus family PHD finger 2 (Pygo2) and ATG14 expression; the PVT1/miR-619-5p/Wnt/β-catenin axis promotes cellular autophagy and attenuates resistance to gemcitabine (Zhou et al., 2020).

### lncRNAs and ferroptosis

Studies examining the involvement of lncRNAs in ferroptosis for the occurrence, development, and metallurgical treatment of HCC, gastric cancer, colon cancer, PCa, kidney cancer, and lung cancer are summarized in Table 5. For example, the high expression of brain-derived neurotrophic factor antisense RNA (BDNF-AS) is positively correlated with the progression and poor prognosis of gastric cancer (Huang G. et al., 2022). BDNF-AS overexpression protects gastric cancer cells from ferroptosis and promotes the progression of gastric cancer. The BDNF-AS/WD repeat domain 5 (WDR5)/F-box and WD repeat domain-containing 7 (FBXW7) axis regulates ferroptosis in gastric cancer cells by affecting the ubiquitination of voltage-dependent anion channel 3 (VDAC3) (Huang G. et al., 2022). CBS mRNA stabilizing lncRNA (CBSLR) interacts with YTH domain linker protein 2 (YTHDF2) to form the CBSLR/YTHDF2/cystathionine beta-synthase (CBS) signal axis, which reduces the stability of CBS mRNA by enhancing the binding of YTHDF2 to the m^6^A-modified coding sequence of CBS mRNA. This protects gastric cancer cells from iron death and leads to resistance to chemotherapy drugs (Yang H. et al., 2022). LINC01606 protects colon cancer cells from ferroptosis by reducing the concentration of iron, lipid ROS, and mitochondrial superoxide, and by increasing mitochondrial membrane potential, while LINC01606 and Wnt/β-catenin form a positive feedback regulatory loop that further inhibits ferroptosis (Luo et al., 2022). lncPVT1 depletion accelerates ferroptosis in HCC cells, and decreased miR-214-3p expression and increased GPX4 expression reverse this effect and promote 4-ketamine-induced ferroptosis (He et al., 2021). In HCC cells, overexpression of nuclear paraspeckle assembly transcript 1 (NEAT1) enhances ferroptosis and the antitumor activity of erastin and RSL3; NEAT1 plays a role in ferroptosis by regulating the expression of miR-362-3p and myo-inositol oxygenase (MIOX) (Zhang Y. et al., 2022). Prostate cancer-associated transcript 1 (PCAT1) suppresses ferroptosis and enhances docetaxel resistance in PCa cells; PCAT1 inhibits the expression of miR-25-3p, thus promoting SLC7A11 expression, while transcription factor AP-2 γ activates PCAT1 expression, and finally reduces ferroptosis and enhances resistance to chemotherapeutic agents (Jiang X. et al., 2022). SLC16A1-antisense nucleic acid 1 (AS1) is highly expressed in kidney cancer, and is associated with overall survival; knockdown of SLC16A1-AS1 expression suppresses the proliferation and migration of kidney cancer cells, and SLC16A1-AS1 induces ferroptosis in kidney cancer through the miR-143-3p/SLC7A11 axis (Li et al., 2022). The metallothionein 1D pseudogene (MT1DP) downregulates miR-365a-3p expression by stabilizing the expression of nuclear factor-erythroid 2 p45-related factor 2, and further promotes ferroptosis in lung cancer cells (Gai et al., 2020). Overexpression of lncRNA H19 eliminates the anticancer effects of curcumin in lung cancer cells, whereas knockdown of H19 expression promotes curcumin-induced ferroptosis; lncRNA H19 acts as a competing endogenous RNA for miR-19b-3p, thereby enhancing the transcriptional activity of its endogenous target, ferritin heavy chain 1 (FTH1) (Zhang R. et al., 2022).

**TABLE 5:** lncRNAs and ferroptosis.

Disease	lncRNA	Mechanism	Ref.
GC	BDNF-AS	The BDNF-AS/WDR5/FBXW7 axis affects VDAC3 ubiquitination to regulate ferroptosis	Huang G. et al. (2022)
	CBSLR	CBSLR interacts with YTHDF2 to form the CBSLR/YTHDF2/CBS axis	Yang H et al. (2022)
CRC	LINC01606	LINC01606 and Wnt/β-catenin form a positive feedback adjustment loop;	Luo et al. (2022)
HCC	PVT1	lncPVT1 depletion accelerates ferroptosis in HCC cells	He et al. (2021)
	NEAT1	NEAT1 regulates miR-362-3p and MIOX expression to promote ferroptosis	Zhang Y. et al. (2022)
PCa	PCAT1	PCAT1 regulates the c-Myc/miR-25-3p/SLC7A11 axis	Jiang X. et al. (2022)
Kidney cancer	SLC16A1-AS1	SLC16A1-AS1 regulates the miR-143-3p/SLC7A11 axis	Li et al. (2022)
Lung cancer	MT1DP	The MT1DP/miR-365a-3p/NRF2 axis improves ferroptosis in NSCLC cells	Cai et al. (2022)
	H19	The H19/miR-19b-3p/FTH1 axis regulates ferroptosis	Zhang R. et al. (2022)

### lncRNAs and pyroptosis

At present, research on the participation of lncRNAs in pyroptosis in tumors focuses mainly on using bioinformatics technology to predict the role of lncRNAs associated with pyroptosis in tumor diagnosis, treatment, and prognosis. However, molecular biology experiments are needed to verify their potential functions. For example, investigators downloaded the sequencing results from a total of 454 lung adenocarcinoma samples from The Cancer Genome Atlas (TCGA) database and identified 19 prognostic lncRNAs related to pyroptosis (Huang H. et al., 2022). Based on RNA sequencing data from the TCGA database, researchers analyzed 14 lncRNAs associated with pyroptosis that may be prognostic markers and promising therapeutic targets in head and neck SCC (Zhu W. et al., 2021). Pyroptosis-associated lncRNAs are associated with the tumor immune microenvironment, which may provide a new indicator for the selection of patients with ovarian cancer for immunotherapy (Zhang et al., 2021e).

According to analyses of the TCGA and China Glioma Genome Atlas databases, a pyroptosis-related lncRNA model was established by using consensus clustering and weighted gene co-expression network analysis, and glioblastoma patients with a high pyroptosis-related lncRNA score were found to have a richer immune infiltrate, higher immune checkpoint gene expression, and better response to immunotherapy, but worse response to chemotherapy (Xing et al., 2022). In gastric adenocarcinoma, HAND2-AS1, LINC01354, RP11-276H19.1, and PGM5-AS1 were suggested to be involved in pyroptosis, which could be used to guide effective patient prognosis and to provide evidence for the development of molecular-targeted therapies associated with pyroptosis (Wang Z. et al., 2022). lncRNA-XIST knockdown triggers pyroptosis mediated by the miR-335/superoxide dismutase 2/ROS signaling pathway, thereby inhibiting the progression of NSCLC (Liu et al., 2019). An lncRNA profile including ELFN1-AS1, PCAT6, TNRC6C-AS1, and ZEB1-AS1 related to pyroptosis could accurately predict the prognosis of CRC patients (Chen S. et al., 2021).

An investigator-constructed risk model containing 10 lncRNAs associated with pyroptosis that were identified as independent predictors of overall survival in breast cancer patients, with RP11-459E5.1, RP11-1070N10.3, and RP11-817J15.3 downregulation, was significantly associated with worse overall survival (Yang et al., 2021d). Eight pyroptosis-associated lncRNAs were identified by using a co-expression network of genes and lncRNAs and by further screening with univariate Cox regression analysis, which may be potential molecular markers and therapeutic targets in patients with bladder cancer (Lia et al., 2022). A novel risk score for pyroptosis-related lncRNAs could be used as a promising prognostic biomarker for HCC patients and may help to guide precision drugs and immunotherapy (Wang T. et al., 2022). Based on transcriptomic data, miRNA sequencing data, and related clinical information downloaded from the TCGA database, and constructed a competing endogenous RNA regulatory network that included 132 lncRNAs and 7 miRNAs, and established 11 lncRNA risk models related to pyroptosis, which has good prognostic value and can predict the immunotherapy outcome of colon adenocarcinoma (Tan et al., 2022).

## Role of circRNAs in tumor cell death for tumorigenesis, development, and treatment

### circRNAs and autophagy

CircRNAs are involved mainly in the influence of autophagy on the occurrence, development, and treatment of breast cancer, SCC, NSCLC, bladder cancer, gastric cancer, AML, cervical carcinoma, osteosarcoma, Bozzolo’s disease, PCa, epithelial ovarian cancer, and pancreatic ductal adenocarcinoma, as summarized in Table 6. For example, circDNMT1 increases the proliferation of breast cancer cells by promoting cell autophagy (Du et al., 2018). Ectopically expressed circDNMT1 promotes the nuclear translocation of p53 and AU-rich element RNA-binding factor 1 (AUF1); the nuclear translocation of p53 induces autophagy, while the nuclear translocation of AUF1 reduces the instability of Dnmt1, ultimately increasing DNMT1 translation (Du et al., 2018). While ciRS-7 is highly expressed in triple-negative breast cancer, it suppresses rapamycin-induced autophagy in esophageal SCC, and miR-1299 promotes rapamycin-induced autophagy in esophageal SCC; ciRS-7 interacts with miR-1299 and regulates epidermal growth factor receptor (EGFR) expression to inhibit autophagy in esophageal SCC cells (Meng et al., 2020). circCUL2 overexpression inhibits the proliferation and autophagy of CRC cells and inhibits CRC progression through the miR-208a-3p/PPP6C signaling pathway (Yang B. L. et al., 2022). circUBAP2 induces autophagy in CRC cells *in vitro* and *in vivo*; circUBAP2 interacts directly with miR-582-5p and regulates the expression of forkhead box protein O1 (FOXO1) to promote CRC progression and metastasis (Chen F. et al., 2021). Silencing of circHIPK3 expression significantly inhibits the proliferation of lung cancer cells and induces autophagy; circHIPK3 has potential clinical value in evaluating the prognosis of lung cancer (Chen et al., 2020). High hsa_circ_0085131 expression is associated with the recurrence of NSCLC; hsa_circ_0085131 interacts with miR-654-5p to promote the expression of ATG7, thus enhancing the resistance of NSCLC cells to cisplatin (Kong, 2020). Reduced hsa_circ_0007813 expression inhibits the proliferation, migration, and autophagy of bladder cancer cells *in vitro* and *in vivo*; hsa_circ_0007813 binds to hsa-miR-361-3p and regulates the expression of insulin-like growth factor 2 receptor, thereby affecting bladder cancer progression (Zhang et al., 2021d). Silencing of circ_0001658 expression reduces the viability and autophagy of gastric cancer cells, and circ_0001658 acts by binding miR-182 and inhibiting the expression of member RAS oncogene family (RAB10) (Duan et al., 2022). circRNA-poly (A)-nuclease deadenylation complex subunit 3 (circPAN3) increases the drug resistance of AML cells by regulating autophagy, and the AMPK/mTOR signaling pathway plays a key role in this process (Shang et al., 2019). Knockdown of circRNA TOPBP1-interacting checkpoint and replication regulator (circTICRR) expression activates autophagy in cervical cancer cells; circTICRR binds to F287/F289 in the ribonucleotide reductase regulatory subunit M3 domain of human antigen R (HuR) and increases the expression of glutamate dehydrogenase 1 (GLUD1) protein, ultimately promoting autophagy (Zhu et al., 2022). circMRPS35 increases microtubule associated protein 1 light chain 3 (LC3) and Beclin-1 expression to promote autophagy in osteosarcoma cells (Jiang C. et al., 2022). After downregulation in osteosarcoma cells, circCRIM1 inhibits cell proliferation, promotes cell autophagy, and acts on the miR-432-5p/HDAC4 axis to inhibit osteosarcoma growth (Liu J. et al., 2020). The hsa_circ_0003489/miR-874-3p/HDAC1 axis balances apoptosis and autophagy in multiple myeloma cells, silences the expression of hsa_circ_0003489, inhibits autophagy, and enhances the sensitivity of multiple myeloma cells to bortezomib (Tian et al., 2021).

**TABLE 6:** circRNAs and autophagy.

Disease	circRNA	Mechanism	Ref.
Breast cancer	circ-Dnmt1	circ-Dnmt1 can interact with p53 and AUF1 to inhibit tumor growth	Du et al. (2018)
ESCC	ciRS-7	The ciRS-7/miR-1299/EGFR axis inhibits esophageal SCC cell autophagy	Meng et al. (2020)
CRC	circCUL2	The circCUL2/miR-208a-3p/PPP6C axis inhibits CRC progression	Yang B. L. et al. (2022)
	circUBAP2	The circUBAP2/miR-582-5p/FOXO1 axis promotes CRC progression and metastasis	Chen F. et al. (2021)
NSCLC	circHIPK3	The circHIPK3/miR124-3p-STAT3-PRKAA/AMPK axis induces autophagy	Chen et al. (2020)
	hsa_circ_0085131	The hsa_circ_0085131/miR-654-5p/ATG7 axis enhances the resistance of NSCLC cells to cisplatin	Kong (2020)
BC	hsa_circ_0007813	The hsa_circ_0007813/hsa-miR-361-3p/IGF2R axis affects autophagy	Zhang et al. (2021d)
GC	circ_0001658	circ_0001658 inhibits RAB10 expression by binding to miR-182	Duan et al. (2022)
AML	circPAN3	The AMPK/mTOR signaling pathway plays a key role in autophagy	Shang et al. (2019)
CC	circTICRR	circTICRR interacts with HuR protein and stabilizes GLUD1 mRNA expression, thereby increasing the level of the GLUD1 protein	Zhu et al. (2022)
Osteosarcoma	circMRPS35	circMRPS35 promotes LC3 and Beclin-1 expression	Jiang C. et al. (2022)
	circCRIM1	The circCRIM1/miR-432-5p/HDAC4 axis regulates the progression of osteosarcoma	Liu J. et al. (2020)
MM	hsa_circ_0003489	The hsa_circ_0003489/miR-874-3p/HDAC1 axis regulates the balance between apoptosis and autophagy	Tian et al. (2021)
CML	circ_0009910	The circ_0009910/miR-34a-5p/ULK1 axis induces autophagy	Cao et al. (2020)
PCa	circ_CCNB2	The circ_CCNB2/miR-30b-5p/KIF18A axis promotes PCa radiosensitivity	Cai et al. (2022)
EOC	circMUC16	circMUC16 binds to miR-199a-5p and relieves its inhibitory effect on Beclin-1 and RUNX1	Gan et al. (2020)
PDAC	circRHOBTB3	The circRHOBTB3/miR-600/NACC1 axis regulates autophagy	Yang et al. (2021c)

Knockdown of circ_0009910 expression inhibits CML cell proliferation and autophagy; the circ_0009910/miR-34a-5p/ULK1 axis induces autophagy in CML cells and accelerates the development of resistance to imatinib (Cao et al., 2020). Knockdown of circ_CCNB2 expression inhibits autophagy in PCa cells and promotes their sensitivity to radiotherapy through the miR-30b-5p/kinesin family member 18A (KIF18A) axis (Cai et al., 2022). circMUC16-mediated autophagy accelerates the invasion and metastasis of epithelial ovarian cancer, and circMUC16 can bind directly to miR-199a-5p and deinhibit the target genes Beclin-1 and Runt-related transcription factor 1 (RUNX1) to exert its effects (Gan et al., 2020). circRHOBTB3 is highly expressed in pancreatic ductal cancer cells, binds directly to miR-600 and regulates the expression of nucleus accumbens-associated 1 (NACC1), thereby promoting the autophagy response mediated by the Akt/mTOR pathway (Yang et al., 2021c).

### circRNAs and ferroptosis

The influence of circRNAs on ferroptosis in the occurrence, development, and treatment of HCC, lung cancer, cervical cancer, thyroid carcinoma, OSCC, breast cancer, glioma, thyroid papillary carcinoma, rectal carcinoma, and acute lymphoblastic leukemia (ALL) is summarized in Table 7. For example, knockdown of circ0097009 expression inhibits the proliferation and invasion of HCC cells; circ0097009, as a competing endogenous RNA, adsorbs miR-1261 and regulates the expression of SLC7A11, a key regulator of ferroptosis, to affect the occurrence and development of liver cancer (Lyu et al., 2021). The expression of cIARS (hsa_circ_0008367) is significantly upregulated in HCC cells after treatment with sorafenib. Downregulation of cIARS expression inhibits ferroptosis, and cIARS inhibits AlkB homolog 5, RNA demethylase (ALKBH5)-mediated autophagy and inhibits sorafenib-induced ferroptosis (Liu Z. et al., 2020). circFOXP1 expression is significantly upregulated in lung cancer tissues, and knockdown of circFOXP1 expression inhibits the viability of lung cancer cells and promotes lung cancer progression by inhibiting ferroptosis (Li and Liu, 2022). circRNA denticleless E3 ubiquitin protein ligase homolog (circDTL) expression is upregulated in NSCLC cells, and knockdown of its expression promotes apoptosis and ferroptosis in NSCLC cells; circDTL functions through the miR-1287-5p/GPX4 axis, which provides a potential target for NSCLC treatment (Shanshan et al., 2021). circLMO1 overexpression *in vitro* and *in vivo* suppresses the growth and metastasis of cervical cancer, and circLMO1 inhibits cervical cancer progression through ferroptosis mediated by the miR-4291/ACSL4 axis (Ou et al., 2022). Knockdown of circ_0067934 expression *in vitro* and *in vivo* induces the proliferation and apoptosis of thyroid cancer cells; circ_0067934 inhibits ferroptosis in thyroid cancer cells through the miR-545-3p/SLC7A11 axis (Wang et al., 2021). Reduced circFNDC3B expression inhibits the growth and ferroptosis of OSCC cells, and the circFNDC3B/miR-520d-5p/SLC7A11 axis promotes the progression of OSCC (Yang et al., 2021a). circRHOT1 acts via the miR-106a-5p/signal transducer and activator of transcription 3 (STAT3) axis to promote the malignant progression of breast cancer and alleviates ferroptosis, providing new insights into the molecular mechanisms underlying the development of breast cancer (Zhang et al., 2021a). In glioma tissues and cells, the expression of the circRNA tau tubulin kinase 2 (circTTBK2) and integrin subunit beta 8 (ITGB8) is upregulated, and circTTBK2 targets ITGB8 expression to promote glioma cell proliferation, invasion, and ferroptosis (Zhang H. Y. et al., 2020). Knockdown of circRNA kinesin family member 4A (circKIF4A) expression inhibits the growth and migration of papillary thyroid cells; the circKIF4A-miR-1231-GPX4 axis plays an important role in promoting the proliferation and ferroptosis of papillary thyroid cancer cells (Chen W. et al., 2021). In rectal cancer tissues, the expression of circRNA ATP-binding box subfamily B member 10 (circABCB10) and C-C motif chemokine ligand 5 (CCL5) is upregulated, while miR-326 is knocked down to promote ferroptosis; the circABCB10/miR-326/CCL5 axis affects rectal cancer cell ferroptosis (Xian et al., 2020). Knockdown of circ_0000745 expression in an ALL cell line suppresses cell cycle progression and glycolysis, and triggers apoptosis and ferroptosis; circ_0000745 binding to miR-494-3p induces neuroepithelial cell transforming 1 (NET1) expression and promotes the development of ALL (Yang X. et al., 2022). circPVT1 enhances the sensitivity of esophageal SCC cells to 5-fluorouracil and regulates the Wnt/β-catenin pathway and ferroptosis via the miR-30a-5p/frizzled class receptor 3 (FZD3) axis (Yao et al., 2021).

**TABLE 7:** circRNAs and ferroptosis.

Disease	circRNA	Mechanism	Ref.
HCC	circ0097009	circ0097009 regulates SLC7A11 expression	Lyu et al. (2021)
	cIARS	cIARS inhibits ALKBH5-mediated autophagy and sorafenib-induced ferroptosis	Liu Z. et al. (2020)
Lung cancer	circFOXP1	NA	Li and Liu (2022)
	circDTL	The circDTL/miR-1287-5p/GPX4 axis inhibits ferroptosis in NSCLC	Shanshan et al. (2021)
Cervical cancer	circLMO1	The circLMO1/miR-4291/ACSL4 axis mediates ferroptosis	Ou et al. (2022)
Thyroid carcinoma	circ_0067934	The miR-545-3p/SLC7A11 axis inhibits ferroptosis	Wang et al. (2021)
OSCC	circFNDC3B	The circFNDC3B/miR-520d-5p/SLC7A11 axis promotes OSCC progression	Yang et al. (2021a)
Breast cancer	circRHOT1	The circRHOT1/miR-106a-5p/STAT3 axis alleviates ferroptosis	Zhang et al. (2021a)
Glioma	circ-TTBK2	circ-TTBK2 regulates ITGB8 expression and promotes ferroptosis	Zhang H. et al. (2020)
TPC	circKIF4A	The circKIF4A/miR-1231/GPX4 axis promotes ferroptosis	Chen W. et al. (2021)
Rectal carcinoma	circABCB10	The circABCB10/miR-326/CCL5 axis promotes ferroptosis	Xian et al. (2020)
ALL	circ_0000745	circ_0000745 binds to miR-494-3p and induces NET1 expression to promote the development of ALL	Yang X. L.et al. (2022)
ECC	circPVT1	The circPVT1/miR-30a-5p/FZD3 axis regulates ferroptosis	Yao et al. (2021)

### circRNAs and pyroptosis

Pyroptosis is an important natural immune response of the body that plays a key role in fighting infection (Deng et al., 2022). circRNAs belong to a class of abundant non-coding RNAs, with stability and specificity, and thus have great potential in cancer treatment (Zhang et al., 2021b). The involvement of circRNAs in the role of pyroptosis in tumors has not been studied in depth. Investigators selected 1,875 differentially expressed circRNAs in unirradiated and irradiated lung cancer A549 cells (Zhang T. et al., 2022). Knockdown of circRNA Nei-like DNA glycosylase 3 (circNEIL3) expression was shown to promote radiation-induced pyroptosis. However, circNEIL3 overexpression had the opposite effect; by binding to miR-1184, circNEIL3 released the inhibitory effect of miR-1184 on PIF1 5′-to-3′ DNA helicase (PIF1) to induce DNA damage and trigger the activation of the absent in melanoma 2 inflammasome. The circNEIL3/miR-1184/PIF1 axis may be a new and promising clinical treatment strategy for lung cancer (Zhang T. et al., 2022).

## Conclusion and future directions

Clinical and basic research into cancer, as a profound public health issue worldwide, has made many breakthroughs in recent years, but we still lack the ability to control tumor morbidity and mortality. How to kill tumor cells accurately and protect normal cells is the last and most important focus of tumor research. With our deepening understanding of intracellular molecules, an increasing number of molecules have been identified with roles in tumor progression, and the participation of non-coding RNAs in cell death plays a key role in tumor occurrence and development. However, under different biological backgrounds, regulated cell death may play very different biological roles. The functions of most cell death-related genes in tumors have not been studied thoroughly, and the signal of regulated cell death in tumors is also undecipherable. Therefore, revealing the regulatory mechanisms of non-coding RNAs involved in cell death in cancer, identifying potential therapeutic cell death targets of cancer, and developing novel immunotherapies based on the non-coding RNAs involved in cell death will have great and far-reaching significance for conquering cancer.

## Figures and Tables

**Table:** 

**β2-MG**	beta-2-microglobulin
**ACSL4**	acyl-CoA synthetase long chain family member 4
**AIM2**	absent in melanoma 2
**ALL**	acute lymphoblastic leukemia
**AML**	acute myelogenous leukemia
**AMPK**	adenosine monophosphate-activated protein kinase
**ATF4**	activated transcription factor 4
**ATG10**	autophagy related 10
**ATG2B**	autophagy related 2B
**ATG5**	autophagy related 5
**AUF1**	AU-rich element RNA-binding factor 1
**CBS**	cystathionine beta-synthase
**CBSLR**	CBS mRNA stabilizing lncRNA
**CDDP**	cisplatin
**CGGA**	China Glioma Genome Atlas
**circRNA**	circular RNA
**circABCB10**	circRNA ATP binding box subfamily B member 10
**circDNMT1**	circRNA DNA methyltransferase 1
**circDTL**	circRNA denticleless E3 ubiquitin protein ligase
**circKIF4A**	circRNA kinesin family member 4A
**circNEIL3**	circRNA nei like DNA glycosylase 3
**circPAN3**	circRNA poly(A)-nuclease deadenylation complex subunit 3
**circTICRR**	circRNA TOPBP1-interacting checkpoint and replication regulator
**circTTBK2**	circRNA tau tubulin kinase 2
**CLRN1-AS1**	CLRN1 antisense RNA 1
**COAD**	colon adenocarcinoma
**CRC**	colorectal cancer
**DDP**	cisplatin
**DEPRG**	differentially expressed pyroptosis-related gene
**DNMT1**	DNA methyltransferase 1
**DMBG**	melbine
**DNMT3B**	DNA methyltransferase 3 beta
**DRAIC**	downregulated RNA in cancer
**EGR1**	early growth response 1
**EIF3J-DT**	EIF3J divergent transcript
**EPHA7**	ephrin type-A receptor 7
**ESCC**	esophageal squamous cell cancer
**EV-A71**	enterovirus A71
**FOXO1**	forkhead box protein O1
**FOXP1**	forkhead box protein P1
**FTH1**	ferritin heavy chain 1
**FZD3**	frizzled class receptor 3
**GAS5**	growth arrest specificity 5
**GBM**	glioblastoma
**GLUT1**	glucose transporter protein 1
**GPX4**	glutathione peroxidase 4
**GSEC**	G-quadruplex forming sequence containing lncRNA
**GSH**	glutathione
**HAGLROS**	HAGLR opposite strand long non-coding RNA
**HNSCC**	head and neck squamous cell carcinoma
**HOTAIR**	HOX transcript antisense RNA
**HOTAIRM1**	HOXA transcript antisense RNA, myeloid-specific 1
**ICB**	immune checkpoint blockade
**IM**	imtatinib
**IREB2**	iron reaction element-binding protein 2
**ISL**	isoliquiritigenin
**KIF18A**	kinesin family member 18A
**LC3**	microtubule associated protein 1 light chain 3
**LDHA**	lactate dehydrogenase A
**lncRNA**	long non-coding RNA
**LOX**	lipoxygenases
**L-ROS**	lipid reactive oxygen species
**LRRK2**	leucine-rich repeat kinase 2
**LUAD**	lung adenocarcinoma
**MAP1S**	microtubule-associated protein 1S
**MATAT1**	metastasis-associated lung adenocarcinoma transcript 1
**MDA**	malondialdehyde
**MDM2**	transformed 3T3 cell double minute 2
**Met**	metformin
**Mig-6**	mitogen-induced gene 6
**MIOX**	myo-inositol oxygenase
**miRNA**	microRNA
**MT1DP**	metallothionein 1D pseudogenes
**mTOR**	mammalian target of rapamycin
**NBAT1**	neuroblastoma-associated transcript 1
**NEAT1**	nuclear paraspeckle assembly transcript 1
**ncRNA**	non-coding
**NET1**	nuroepithelial cell transforming 1
**NLRP3**	nucleotide-binding oligomerization domain-like receptor protein 3
**NPC**	nasopharyngeal carcinoma
**NQO1**	NAD(P)H quinone dehydrogenase 1
**NRF2**	nuclear factor erythroid 2-related factor 2
**OIP5-AS1**	opa interacting protein 5 antisense RNA 1
**OSSC**	oral squamous carcinoma
**PCa**	prostate cancer
**PCDRlnc1**	PCa docetaxel resistance-related lncRNA 1
**PEM**	pemetrexed
**PRL**	pyroptosis-related lncRNA
**PTC**	papillary thyroid carcinoma
**Pygo2**	Pygopus family PHD finger 2
**RAB10**	member RAS oncogene family
**RCC**	renal cell carcinoma
**RCD**	regulated cell death
**RELN**	recombinant reelin
**ROS**	reactive oxygen species
**RSL3**	RAS-selective lethal 3
**SLC1A5**	solute carrier family 1 member 5
**SLC16A1-AS1**	solute carrier family 16 member 1-antisense nucleic acid 1
**SLC7A11**	solute carrier family 7 member 11
**SNHG1**	small nucleolar RNA host gene 1
**SNHG15**	small nucleolar RNA host gene 15
**SNHG7**	small nucleolar RNA host gene 7
**STAT3**	signal transducer and activator of transcription 3
**TCGA**	The Cancer Genome Atlas
**TFR1**	transferrin receptor 1
**TGF-β**	transforming growth factor beta 1
**TNBC**	triple-negative breast cancer
**TPT1**	tumor protein translational control 1
**TPT1-AS**	TPT1 antisense RNA 1
**UCA1**	urothelial carcinoma associated with 1
**UHRF1**	ubiquitin-like with plant homeodomain and ring finger domain 1
**ULK1**	Unc-51 autophagy-activated kinase 1
**ULK3**	Unc-51-like kinase 3
**UM**	uveal melanoma
**VDAC3**	voltage dependent anion channel 3
**WGCNA**	weighted gene co-expression network analysis
**YTHDF2**	YTH domain linker protein 2
**ZNNT1**	ZNF706 neighboring transcript 1
**ESCC**	esophageal SCC

## Supplementary material

SUPPLEMENTARY FIGURE S1 Schematic representation of the regulatory mechanism of autophagy.

SUPPLEMENTARY FIGURE S2 Schematic representation of the regulatory mechanisms of ferroptosis.

SUPPLEMENTARY FIGURE S3 Schematic representation of the regulatory mechanisms of autophagy.
