# Supplementary figures and images for: Non-coding RNAs are involved in tumor cell death and affect tumorigenesis, progression, and treatment: a systematic review
Source: Front Cell Dev Biol. 2024 Feb 28;12:1284934. doi: 10.3389/fcell.2024.1284934 (PMC10936223; doi:10.3389/fcell.2024.1284934)

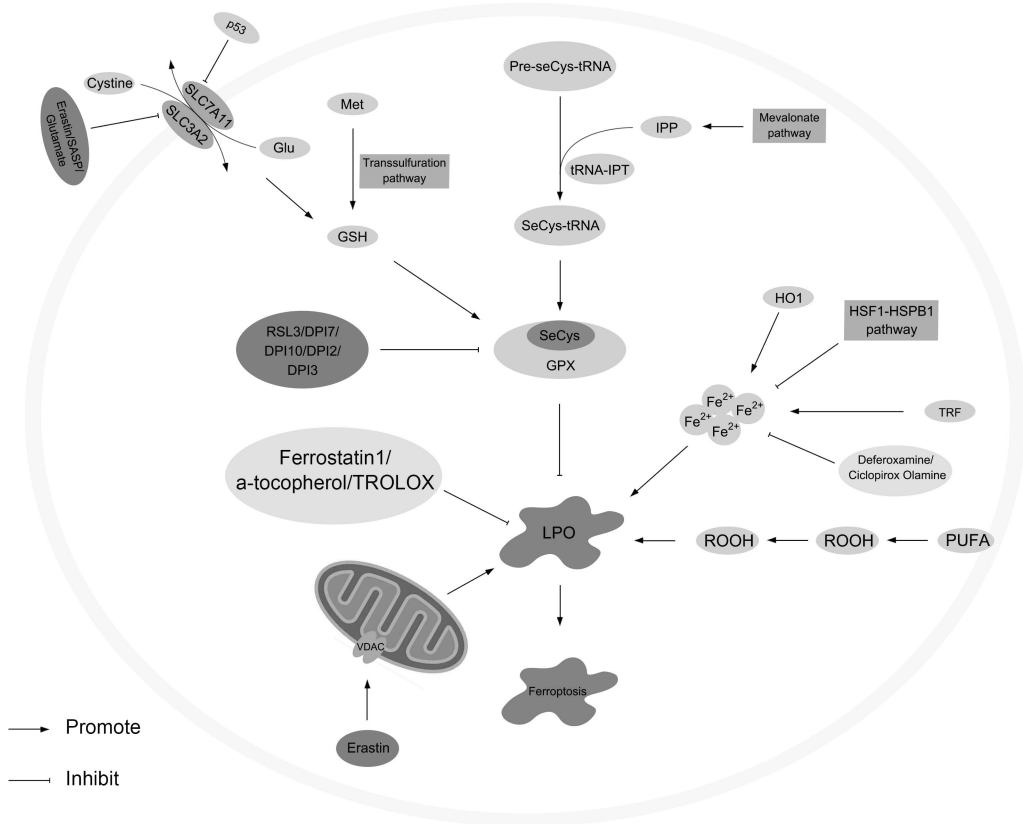

Supplement: Supplementary file 1 [file Image2.pdf]

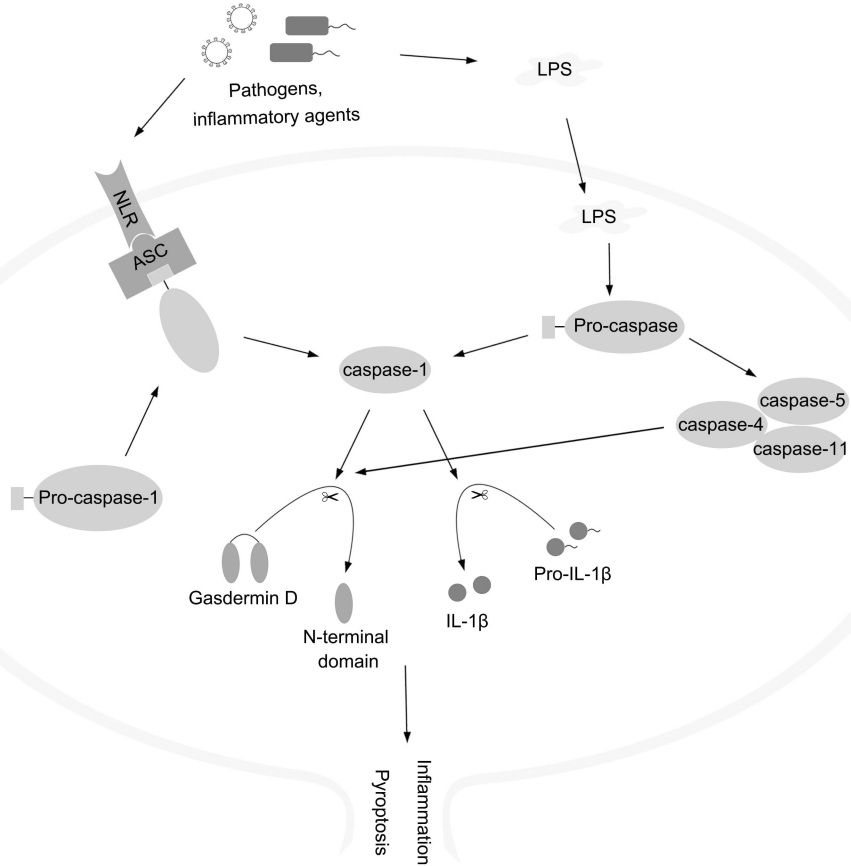

Supplement: Supplementary file 2 [file Image3.pdf]

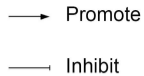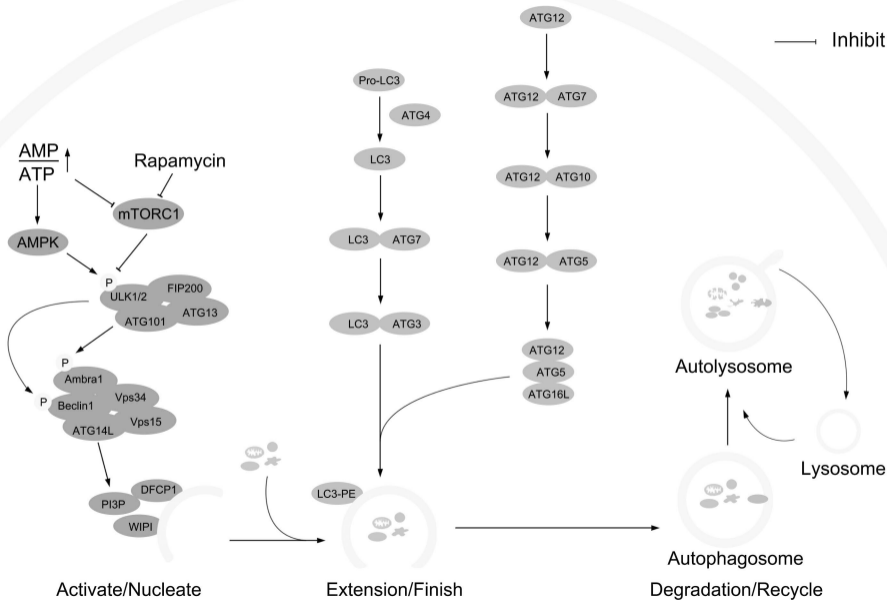

Supplement: Supplementary file 4 [file Image1.pdf]
