# Supplementary material for: Non-coding RNAs are involved in tumor cell death and affect tumorigenesis, progression, and treatment: a systematic review
Source: Front Cell Dev Biol. 2024 Feb 28;12:1284934. doi: 10.3389/fcell.2024.1284934 (PMC10936223; doi:10.3389/fcell.2024.1284934)
Supplement: Supplementary file 3 [file Table1.doc]

Supplementary Table 1. Comparison of the characteristics of autophagy, ferroptosis, and pyroptosis

|  | **Autophagy** | **Ferroptosis** | **Pyroptosis** |
| --- | --- | --- | --- |
| **Characteristic** | Programmed death | Programmed death | Programmed death |
| **Inducing agent** | Sodium, carbamazepine, and valproate | RSIS, sorafenib, FIN56, and artemisinins | ZnO.NPs and ivermectin |
| **Cell morphology** | Double-membrane structure wraps the autophagosome to form a crescent-shaped vacuole | Mitochondrial atrophy, mitochondrial membrane rupture, and normal nucleus | Cell swelling, multiple pores appear in the cell membrane, and pyroptotic bodies are formed |
| **Cell membrane** | Structural integrity | Rupture | Rupture |
| **Key biochemical features** | ATG family proteins are involved in autophagy-lysosomal encapsulation and degradation | Intracellular Fe^2+^ accumulation, ROS accumulation, and glutathione reduction | NLRP3 and other inflammasomes activate caspase-1 |
| **DNA** | Random degradation | Random degradation | Random degradation |
| [**Classification**](javascript:;) | Microautophagy;macroautophagy;Chaperone-mediated autophagy | NA | NA |
