# Supplementary material for: Non-coding RNAs are involved in tumor cell death and affect tumorigenesis, progression, and treatment: a systematic review
Source: Front Cell Dev Biol. 2024 Feb 28;12:1284934. doi: 10.3389/fcell.2024.1284934 (PMC10936223; doi:10.3389/fcell.2024.1284934)
Supplement: Supplementary file 5 [file Table2.doc]

Supplementary Table 2. Comparison of the biological characteristics of miRNAs, lncRNAs, and circRNAs

|  | **miRNA** | **lncRNA** | **circRNA** |
| --- | --- | --- | --- |
| **Structural features** | Precursors capable of forming intramolecular stem-loop structures | mRNA like structures | Closed ring structure |
| **Length** | 21–23 nucleotides | >200 nucleotides | 200–500 nucleotides |
| **Classification** | NA | Sense, antisense, bidirectional, intronic, and intergenic | Exonic, intronic , and retained-intronic |
| **Function** | mRNA degradation, translational inhibition, and transcriptional regulation | Epitranscriptional regulation, genetic regulation, and post-transcriptional regulation | Transcriptional or post-transcriptional regulation; a few can be translated into peptides |
| **Location** | Cell nucleus and cytoplasm | Distributed in the nucleus, cytoplasm, and organelles | In the cytoplasm of eukaryotic cells, circRNAs are mainly derived from exons, and a small amount of circRNAs derived from introns are present in the nucleus |
| **Biological properties** | Highly conserved and temporal- and tissue-specific | Tissue-specific and spatio-temporal | Expression levels are species-, tissue-, and temporal-specific |
